# Best Papers of 2013

**DOI:** 10.1120/jacmp.v15i4.5091

**Published:** 2014-05-21

For the past several years, the *JACMP* has issued awards to several manuscripts deemed by the Board of Editors to be worthy of the designation “Best Papers.” These awards are selected by a committee of the Board of Editors and are supported by the American Association of Physicists in Medicine (AAPM). I offer my sincere congratulations to the authors of these outstanding contributions to the body of knowledge that comprises clinical medical physics.

## Medical Imaging Physics Award

Yamak D, Pavlicek W, Boltz T, Panse P, Akay M. Coronary calcium quantification using contrast‐enhanced dual‐energy computed tomography scans. J Appl Clin Med Phys. 2013;14(3). http://www.jacmp.org/index.php/jacmp/article/view/4014/2902

## Radiation Oncology Physics Award

Shang Q, Sheplan Olsen L, Stephans K, Tendulkar R, Xia P. Prostate rotation detected from implanted markers can affect dose coverage and cannot be simply dismissed. J Appl Clin Med Phys. 2013;14(3). http://www.jacmp.org/index.php/jacmp/article/view/4262/2885

## Radiation Measurements Award

Zhang A, Wen N, Nurushev T, Burmeister J, Chetty I. Comprehensive evaluation and clinical implementation of commercially available Monte Carlo dose calculation algorithm. J Appl Clin Med Phys. 2013;14(2). http://www.jacmp.org/index.php/jacmp/article/view/4062/2838

It is the intent to have one “Best Paper” award winner, the Editor‐in‐Chief Award, invited to the podium during the Awards and Honors Ceremony of the Annual Meeting of the AAPM to accept the award. (In consideration of time, the others will be acknowledged in the program.)

## Editor‐in‐Chief Award

Beyer G. Commissioning measurements for photon beam data on three TrueBeam linear accelerators, and comparison with Trilogy and Clinac 2100 linear accelerators. J Appl Clin Med Phys. 2013;14(1). http://www.jacmp.org/index.php/jacmp/article/view/4077/2780

By listing the Best Papers of 2013, I would like to honor the award winners for their contributions to the clinical practice of medical physics, and thank the AAPM for its financial support.

Michael D. Mills, PhD

Editor‐in‐Chief

